# Photosynthetic and Antioxidant Responses of *Lolium perenne* to Aluminum Oxide Nanoparticles, Their Supernatant, and Aluminum Chloride Under Acidic Conditions

**DOI:** 10.3390/molecules31203708

**Published:** 2026-10-16

**Authors:** Jorge González-Villagra, Valentina Figueroa, Paz Cárcamo-Fincheira, Marjorie Reyes-Díaz, Gonzalo Tortella Fuentes, Adalberto Benavides-Mendoza, Antonio Juárez-Maldonado, Ricardo Tighe-Neira, Claudio Inostroza-Blancheteau

**Affiliations:** 1 Facultad de Ciencias Agrarias, Universidad de Talca, Campus Lircay, Talca 3460000, Chile; jorge.gonzalez@umayor.cl; 2 Departamento de Ciencias Agropecuarias y Acuícolas, Facultad de Recursos Naturales, Universidad Católica de Temuco, Temuco P.O. Box 15-D, Chile; valentinafigueroaboggie@gmail.com; 3 Núcleo de Investigación en Biotecnología y Agroinnovación (NIBA), Escuela de Agronomía, Facultad de Medicina Veterinaria y Agronomía, Universidad de Las Américas, Santiago 7500975, Chile; mcarcamo@udla.cl; 4 Departamento de Ciencias Químicas y Recursos Naturales, Facultad de Ingeniería y Ciencias, Universidad de La Frontera, Temuco P.O. Box 54-D, Chile; marjorie.reyes@ufrontera.cl; 5 Center of Plant, Soil Interaction and Natural Resources Biotechnology, Scientific and Technological Bioresource Nucleus (BIOREN), Universidad de La Frontera, Temuco P.O. Box 54-D, Chile; 6 Centro de Excelencia en Investigación Biotecnológica Aplicada al Medio Ambiente (CIBAMA), Facultad Ingeniería y Ciencias, Universidad de La Frontera, Temuco P.O. Box 54-D, Chile; 7 Departamento de Horticultura, Universidad Autónoma Agraria Antonio Narro, Saltillo 25315, Mexico; adalberto.benavides@uaaan.edu.mx; 8 Departamento de Botánica, Universidad Autónoma Agraria Antonio Narro, Saltillo 25315, Mexico; antonio.juarez@uaaan.edu.mx; 9 Núcleo de Investigación en Producción Alimentaria, Facultad de Recursos Naturales, Universidad Católica de Temuco, Temuco P.O. Box 15-D, Chile; 10 Laboratorio de Fisiología y Biotecnología Vegetal, Facultad de Recursos Naturales, Universidad Católica de Temuco, Temuco P.O. Box 15-D, Chile; 11 ANID–Millennium Science Initiative Program–Millennium Nucleus in Data Science for Plant Resilience (Phytolearning), Santiago 8370186, Chile

**Keywords:** chlorophyll fluorescence, CO_2_ assimilation, oxidative stress, aluminum nanoparticles

## Abstract

Aluminum oxide nanoparticles (Al_2_O_3_ NPs) are frequently incorporated into fertilizers and pesticides. Nevertheless, the effects of Al_2_O_3_ NPs on plants growing in acidic soils with elevated aluminum toxicity remain poorly understood. In this context, we evaluated the effects of aluminum oxide nanoparticles (Al_2_O_3_ NPs) on photosynthetic activity, gene expression, and antioxidant defense mechanisms in the *Lolium perenne* genotype Halo-AR1 cultivated in nutrient solution under acidic conditions. Thus, plants were subjected to Al_2_O_3_ NPs, Al_2_O_3_ NP supernatant, and AlCl_3_ treatments. Photosynthetic performance was significantly impaired in all treatments, as reflected by reduced CO_2_ assimilation, stomatal conductance, effective PSII quantum yield of PSII (ΦPSII), and electron transport rate (ETR), with stronger effects in the Al_2_O_3_ NP supernatant and AlCl_3_ treatments. Furthermore, Al_2_O_3_ NPs and AlCl_3_ downregulated the expression of the photosynthesis-related genes *psbA* and *rbcS* after 48 h of exposure. Antioxidant activity, total phenols, and flavonoid contents increased in leaves and roots, indicating activation of antioxidant defenses, particularly under Al_2_O_3_ NPs and Al_2_O_3_ NP supernatant exposure. Overall, the Al_2_O_3_ NP supernatant and AlCl_3_ treatments produced more pronounced effects on photosynthetic performance than the Al_2_O_3_ NP suspension under the tested conditions. However, the contributions of particulate and dissolved Al to these responses remain unresolved.

## 1. Introduction

Nanotechnology has become an important area of research in modern agriculture, with engineered nanoparticles (NPs) investigated for agricultural applications to improve crop productivity and resource-use efficiency [1,2,3,4,5]. Among the most widely produced metal-based nanomaterials are carbon nanotubes, zinc oxide, titanium dioxide, and aluminum oxide nanoparticles (Al_2_O_3_ NPs) [6]. In particular, Al_2_O_3_ NPs are increasingly used as components of agricultural formulations, raising concerns about their potential environmental fate and biological effects following their release into agricultural systems [7,8].

Aluminum (Al) is the third-most-abundant element in the Earth’s crust and, under acidic conditions (pH ≤ 5.5), is solubilized mainly as phytotoxic Al^3+^, which inhibits root growth, nutrient uptake, and ultimately reduces crop productivity [9,10,11]. Because acidic soils represent nearly 50% of the world’s potentially arable land, the application of Al_2_O_3_ nanoparticles to these environments raises an important question: whether phytotoxic responses are caused primarily by the nanoparticles themselves or by soluble aluminum species released through nanoparticle dissolution under acidic conditions. Addressing this distinction is essential for accurately assessing the environmental risks associated with Al_2_O_3_ nanoparticle use in agriculture. 

Although Al_2_O_3_ nanoparticles have traditionally been regarded as particulate contaminants, their behavior in acidic environments is strongly influenced by dissolution processes that release soluble aluminum species [12,13]. Consequently, the phytotoxic effects reported for Al_2_O_3_ NPs may result from the nanoparticles themselves, from dissolved Al ions, or from the combined action of both fractions. Therefore, distinguishing between particle-mediated and dissolution-mediated phytotoxicity is essential for improving our understanding of the environmental risks associated with Al_2_O_3_ nanoparticle applications in agriculture.

Perennial ryegrass (*Lolium perenne* L.) is one of the most important forage species in temperate agricultural systems, sustaining beef and dairy production in southern Chile [14,15,16]. *L. perenne* is predominantly cultivated on Andisols, which are frequently characterized by low pH ( ≤ 5.5) and high aluminum availability [14]. Consequently, aluminum toxicity represents a major constraint to forage productivity, persistence, and nutritional quality in these production systems.

Previous studies have shown that exposure to aluminum nanoparticles or aluminum ions can impair photosynthesis at both the physiological and molecular levels. For example, in *Arabidopsis thaliana*, aluminum nanoparticles markedly reduced the expression of the photosynthesis-related genes *psbA* and *rbcL*, suggesting impaired carbon fixation [17]. Similarly, our previous work demonstrated differential expression of *psbA*, *rbcL*, and *rbcS* in *Solanum lycopersicum* exposed to TiO_2_ nanoparticles [18]. These findings highlight the importance of integrating physiological and molecular approaches to better understand the mechanisms underlying nanoparticle-induced phytotoxicity.

Therefore, this study aimed to evaluate the effects of an aluminum oxide nanoparticle (Al_2_O_3_ NP) suspension, its centrifugation-derived supernatant, and an AlCl_3_ solution on photosynthetic performance, photosynthesis-related gene expression, and antioxidant responses in *Lolium perenne* grown under acidic conditions.

## 2. Results

The Al_2_O_3_ NPs were < 50 nm in size, as measured by the manufacturer using transmission electron microscopy (TEM). Our measurements in aqueous media using a Z-sizer showed a mean hydrodynamic diameter of 216.8 nm and a polydispersity index (PDI) of 0.226 at 25.1 °C. The measured zeta potential was +32.7 mV.

### 2.1. Chlorophyll Fluorescence of PSII and Gas Exchange

**Figure 1:**
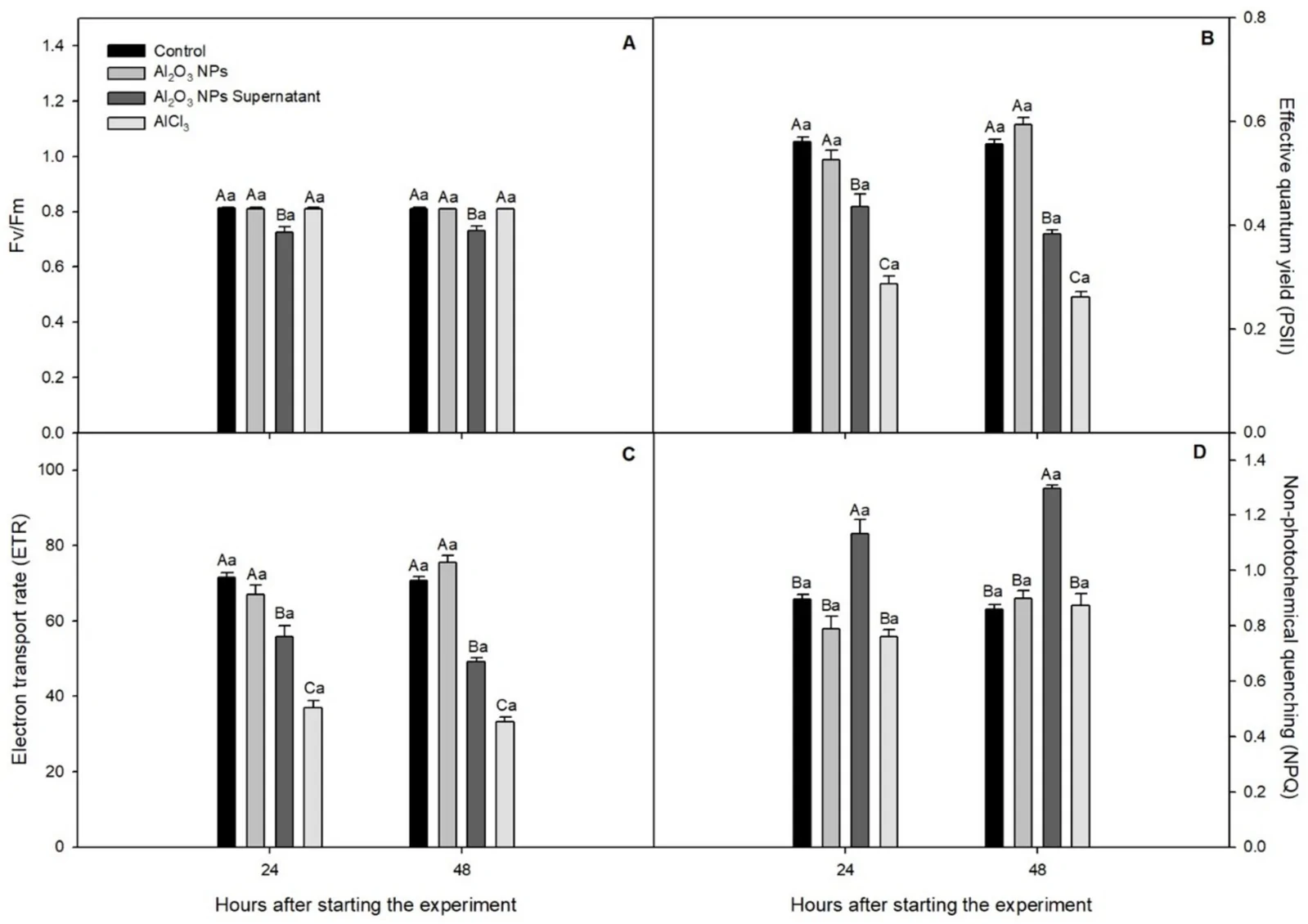
Chlorophyll fluorescence of PSII in perennial ryegrass (*L. perenne*) exposed to: (1) control (without NPs), (2) 500 mg L^−1^ Al_2_O_3_ NPs, (3) 500 mg L^−1^ Al_2_O_3_ NP supernatant, and (4) 200 μM AlCl_3_ for 24 and 48 h. (**A**) The maximum photochemical efficiency of PSII (Fv/Fm), (**B**) effective quantum yield of the PSII, (**C**) electron transport rate, and (**D**) non-photochemical quenching. Different capital letters indicate significant differences among treatments at the same exposure time according to Tukey’s test (*p* ≤ 0.05). Different lowercase letters indicate significant differences in exposure time (24 to 48 h) for the same treatment, as determined by the paired samples Student’s *t*-test (*p* ≤ 0.05).

In our study, the maximum photochemical efficiency of PSII (Fv/Fm) showed no significant differences among treatments during the experiment in *L. perenne*, except for the Al_2_O_3_ NP supernatant, which decreased by 10% compared to the control treatment at 24 and 48 h (Figure 1A). On the other hand, we observed that Al_2_O_3_ NP supernatant treatment significantly decreased 22% and 27% the effective quantum yield of PSII (ΦPSII) in *L. perenne* at 24 and 48 h, respectively, compared to control plants (Figure 1B). Likewise, the electron transport rate (ETR) showed similar tendency to ΦPSII, where *L. perenne* plants decreased their levels under Al_2_O_3_ NP supernatant at 24 and 48 h, showing a decrease of 22% and 32%, respectively, compared to control plants (Figure 1C). Otherwise, AlCl_3_ treatment decreased 48% and 54% the effective quantum yield of PSII (ΦPSII) in *L. perenne* at 24 and 48 h, compared to control plants (Figure 1B). Meanwhile, the electron transport rate (ETR) decreased 48% and 53% at 24 and 48 h, respectively, in *L. perenne* subjected to AlCl_3_ treatment compared to control plants (Figure 1C). Regarding non-photochemical quenching (NPQ), the Al_2_O_3_ NP supernatant treatment showed the highest NPQ values at both 24 and 48 h, compared with all other treatments (Figure 1D). 

**Figure 2:**
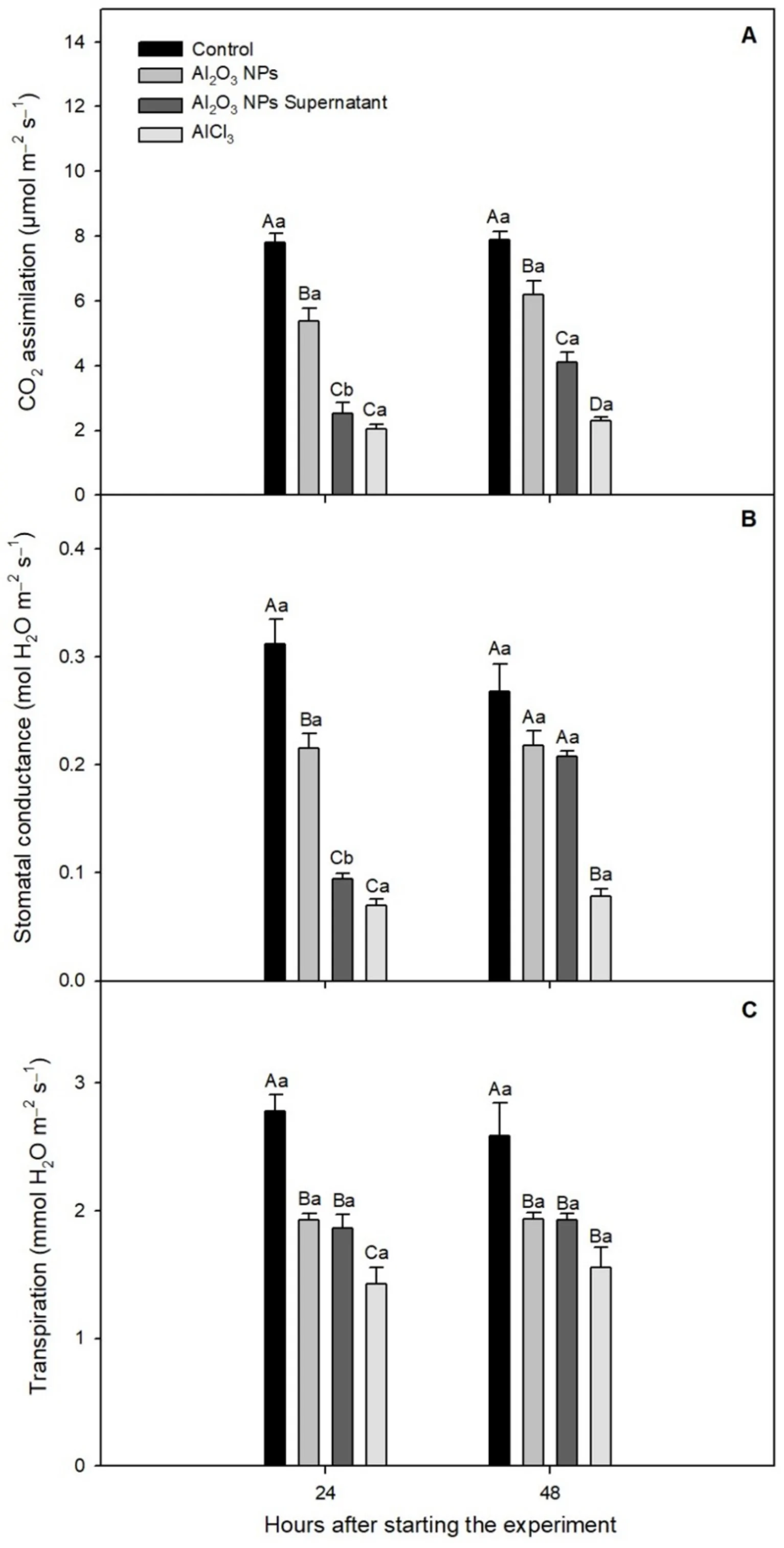
(**A**) CO_2_ assimilation, (**B**) stomatal conductance, and (**C**) transpiration of perennial ryegrass (*L. perenne*) exposed to: (1) control (without NPs), (2) 500 mg L^−1^ Al_2_O_3_ NPs, (3) 500 mg L^−1^ Al_2_O_3_ NP supernatant, and (4) 200 μM AlCl_3_ for 24 and 48 h. Different capital letters indicate significant differences among treatments at the same exposure time according to Tukey’s test (*p* ≤ 0.05). Different lowercase letters indicate significant differences in exposure time (24 to 48 h) for the same treatment, as determined by the paired samples Student’s *t*-test (*p* ≤ 0.05).

On the other hand, our study revealed that CO_2_ assimilation was significantly decreased in all treatments compared to control plants, decreasing by 28%, 58%, and 50% in Al_2_O_3_ NPs, Al_2_O_3_ NP supernatant, and AlCl_3_ treatments, respectively (Figure 2A). Similar tendency was observed in stomatal conductance (gs), where it was reduced in plants subjected to Al_2_O_3_ NPs, Al_2_O_3_ NP supernatant, and AlCl_3_ treatments (Figure 2B). Interestingly, plants under Al2O3 NP supernatant showed an increase in CO_2_ assimilation (by 25%) and gs (by 50%) at 48 h compared to 24 h of the experiment. Concerning transpiration (E), *L. perenne* plants exposed to Al_2_O_3_ NPs and Al_2_O_3_ NP supernatant exhibited transpiration rates approximately 25% lower than the control at both exposure times (Figure 2C). At 24 h, the AlCl_3_ treatment showed the lowest transpiration rate. In contrast, at 48 h no significant differences were detected among the three Al treatments. No significant differences were observed between 24 and 48 h across any treatment.

### 2.2. Gene Expression of Subunits of PSII and Rubisco

**Figure 3:**
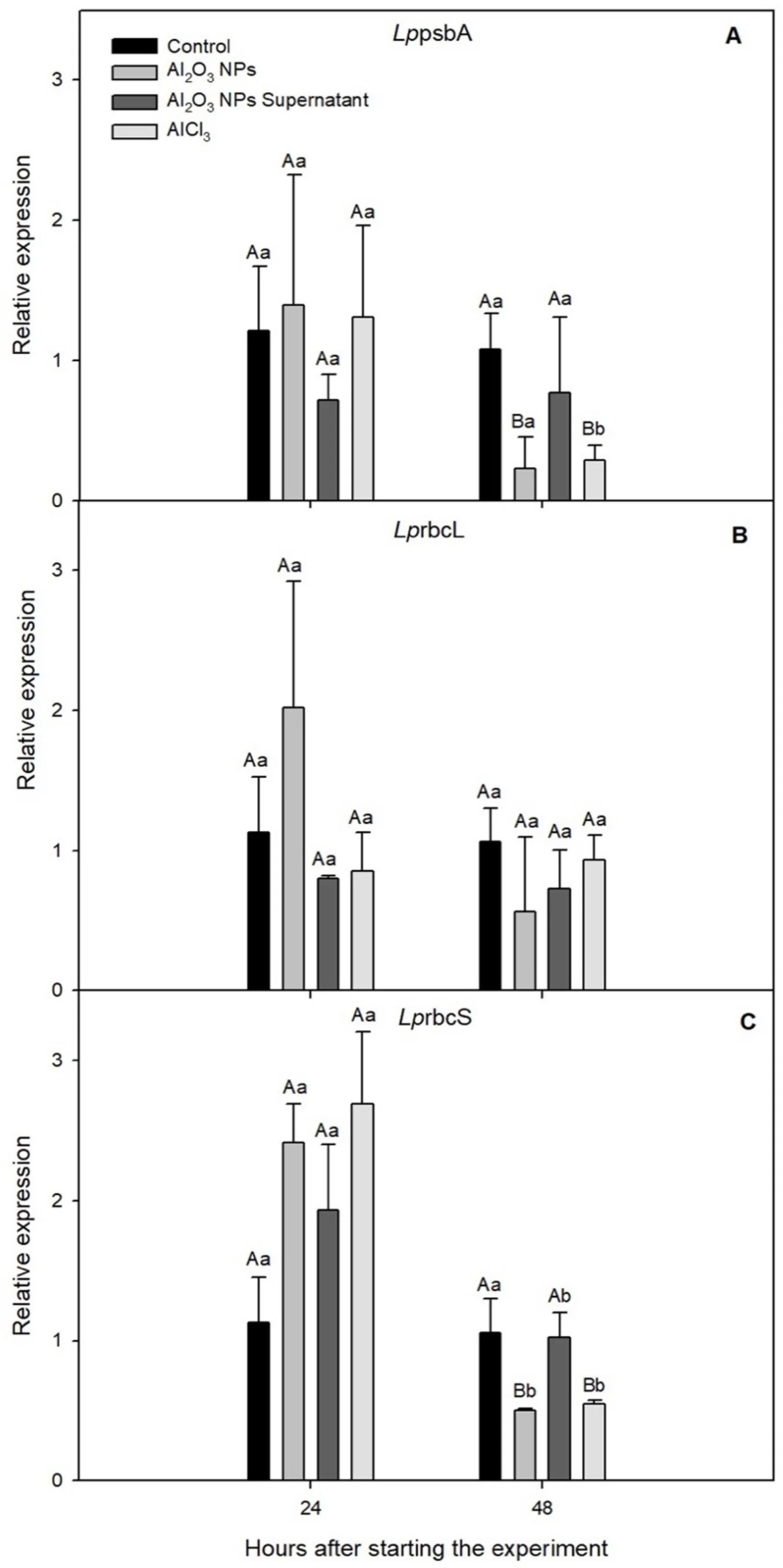
Gene expression of subunits of PSII and Rubisco (**A**) *psbA*, (**B**) *rbcL*, and (**C**) *rbcS* of perennial ryegrass (*L. perenne*) exposed to: (1) control (without NPs), (2) 500 mg L^−1^ Al_2_O_3_ NPs, (3) 500 mg L^−1^ Al_2_O_3_ NP supernatant, and (4) 200 μM AlCl_3_ for 24 and 48 h. Different capital letters indicate significant differences among treatments at the same exposure time according to Tukey’s test (*p* ≤ 0.05). Different lowercase letters indicate significant differences in exposure time (24 to 48 h) for the same treatment, as determined by the paired samples Student’s *t*-test (*p* ≤ 0.05).

In our study, differential gene expression of *psbA*, *rbcL*, and *rbcS* was induced by Al_2_O_3_ NPs, Al_2_O_3_ NP supernatant, and AlCl_3_ treatments (Figure 3). Gene expression of *psbA* did not differ among treatments at 24 h. However, we observed that Al_2_O_3_ NPs and AlCl_3_ treatments significantly reduced (by about 60%) *psbA* gene expression compared to the control treatment at 48 h (Figure 3A). The *rbcL* gene expression did not differ significantly among treatments at either 24 or 48 h, although the Al_2_O_3_ NP treatment showed the highest mean expression at 24 h and lower expression at 48 h (Figure 3B). The *rbcS* gene expression did not differ significantly among treatments at 24 h. At 48 h, Al_2_O_3_ NPs and AlCl_3_ significantly reduced *rbcS* expression compared with the control and the Al_2_O_3_ NP supernatant treatment. In addition, *rbcS* expression decreased significantly from 24 to 48 h in the Al_2_O_3_ NPs, Al_2_O_3_ NP supernatant, and AlCl_3_ treatments, whereas no temporal differences were observed in the control (Figure 3C). 

### 2.3. Photosynthetic Pigments in L. perenne

Regarding photosynthetic pigments, Chl *a*, Chl *b*, Chl *a*+*b*, and Chl *a*/*b* did not vary among treatments at 24 h (Table 1). By contrast, at 48 h into the experiment, *L. perenne* plants treated with Al_2_O_3_ NPs showed the lowest Chl *a* level, 21% lower than in control plants. Regarding Chl *b*, the supernatant from Al_2_O_3_ NPs increased by more than 50% compared to the control plants at 48 h, reaching the highest level among the treatments. A similar trend was observed for Chl *a*+*b*, with the Al_2_O_3_ NP supernatant showing the highest value at 48 h, whereas Al_2_O_3_ NPs resulted in the lowest Chl *a*+*b* content among the treatments (Table 1). In general, no changes in Chl *a* pigment were observed across exposure times for all treatments, except for Al_2_O_3_ NPs, which reduced Chl *a* by about 21% at 48 h compared to 24 h. Concerning Chl *b*, the Al_2_O_3_ NP supernatant treatment showed a significant increase of approximately 171% at 48 h compared with 24 h, whereas no temporal changes were observed in the remaining treatments. A similar pattern was observed for Chl *a*+*b*, which also increased significantly in the Al_2_O_3_ NP supernatant treatment between 24 and 48 h. By contrast, Chl *a* declined significantly only in the Al_2_O_3_ NP treatment over time, while the Chl *a*/*b* ratio decreased significantly in the Al_2_O_3_ NP supernatant treatment and increased in the Al_2_O_3_ NP treatment at 48 h (Table 1).

### 2.4. Antioxidant-Related Parameter Responses in L. perenne

**Figure 4:**
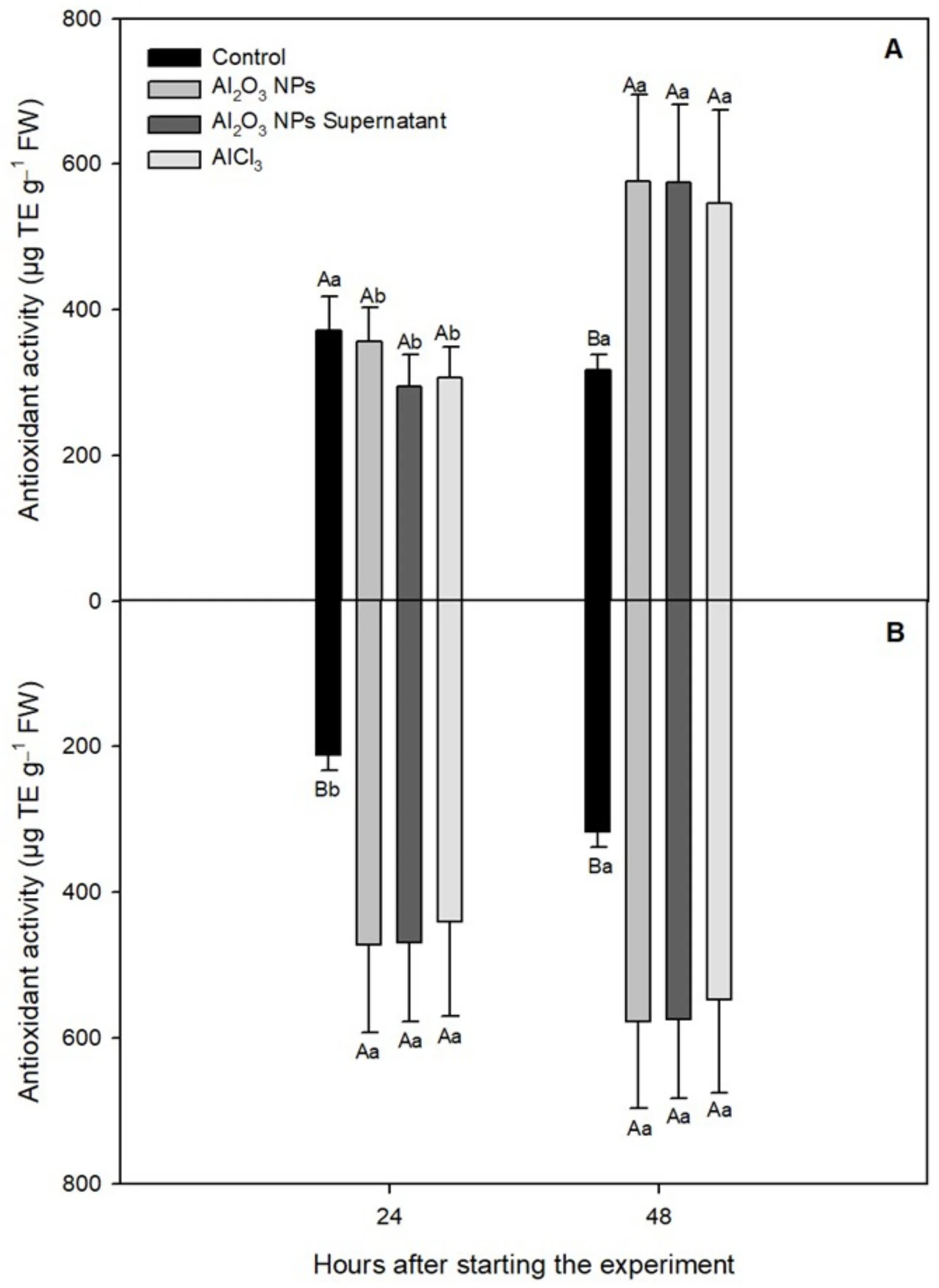
Antioxidant activity in (**A**) leaves and (**B**) roots of perennial ryegrass (*L. perenne*) exposed to: (1) control (without NPs), (2) 500 mg L^−1^ Al_2_O_3_ NPs, (3) 500 mg L^−1^ Al_2_O_3_ NP supernatant, and (4) 200 μM AlCl_3_ for 24 and 48 h. Different capital letters indicate significant differences among treatments at the same exposure time according to Tukey’s test (*p* ≤ 0.05). Different lowercase letters indicate significant differences in exposure time (24 to 48 h) for the same treatment, as determined by the paired samples Student’s *t*-test (*p* ≤ 0.05).

Antioxidant activity (AA) in leaves did not differ among treatments after 24 h of exposure (Figure 4A). However, after 48 h, all treatments (Al_2_O_3_ NPs, Al_2_O_3_ NP supernatant, and AlCl_3_) showed significantly higher AA than the control, with increases of approximately 40%. At the same time, no differences were detected among the three treatments. Temporal analysis showed that AA remained unchanged in control plants but increased significantly from 24 to 48 h in all treatments.

In roots, all treatments exhibited significantly higher AA than the control at both 24 and 48 h, reaching values approximately 2.1-fold greater than those of the control plants (Figure 4B). Similar to leaves, AA remained unchanged over time in the control. In contrast, all treatments showed a significant increase between 24 and 48 h. 

**Figure 5:**
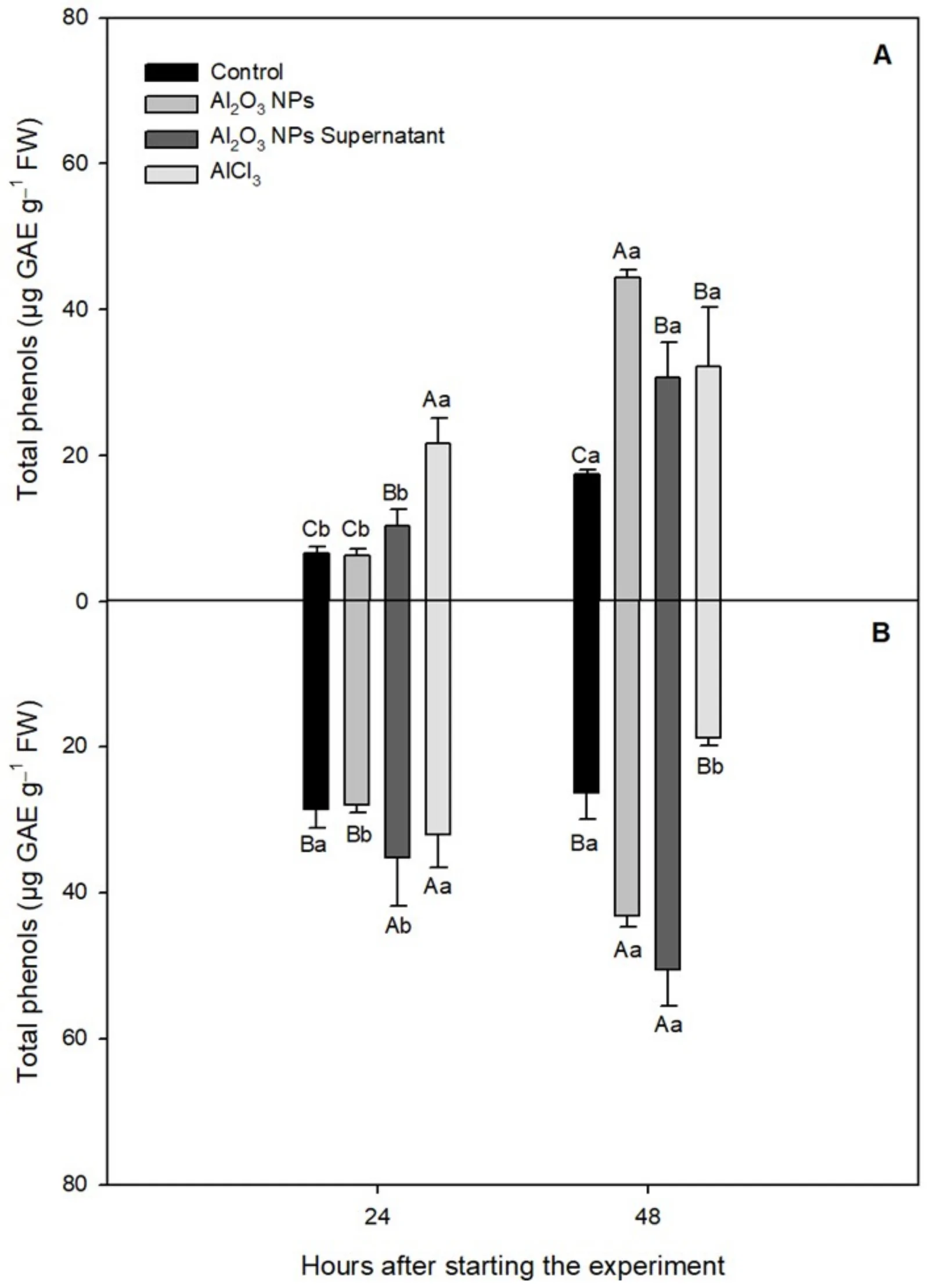
Total phenols in (**A**) leaves and (**B**) roots of perennial ryegrass (*L. perenne*) exposed to: (1) control (without NPs), (2) 500 mg L^−1^ Al_2_O_3_ NPs, (3) 500 mg L^−1^ Al_2_O_3_ NP supernatant, and (4) 200 μM AlCl_3_ for 24 and 48 h. Different capital letters indicate significant differences among treatments at the same exposure time according to Tukey’s test (*p* ≤ 0.05). Different lowercase letters indicate significant differences in exposure time (24 to 48 h) for the same treatment, as determined by the paired samples Student’s *t*-test (*p* ≤ 0.05).

Total phenol (TP) content in leaves differed among treatments at both exposure times (Figure 5A). At 24 h, AlCl_3_ induced the highest TP content, reaching values approximately 3.2-fold higher than the control. In contrast, the Al_2_O_3_ NP supernatant showed an intermediate response, and Al_2_O_3_ NPs did not differ from the control. After 48 h, TP content further increased under all Al treatments, with Al_2_O_3_ NPs exhibiting the highest values (approximately 2.5-fold higher than the control), followed by the Al_2_O_3_ NP supernatant and AlCl_3_, while the control remained the lowest. No significant temporal differences in TP content were detected in leaves within any treatment.

In roots, AlCl_3_ induced the highest TP content at 24 h, whereas Al_2_O_3_ NPs and the Al_2_O_3_ NP supernatant showed intermediate values and the control remained the lowest (Figure 5B). After 48 h, TP content increased further in the Al_2_O_3_ NPs and Al_2_O_3_ NP supernatant treatments, which showed the highest values among treatments, whereas AlCl_3_ exhibited intermediate levels. Temporal analysis revealed a significant increase in the Al_2_O_3_ NP supernatant treatment only between 24 and 48 h, while the remaining treatments did not show significant changes over time. 

**Figure 6:**
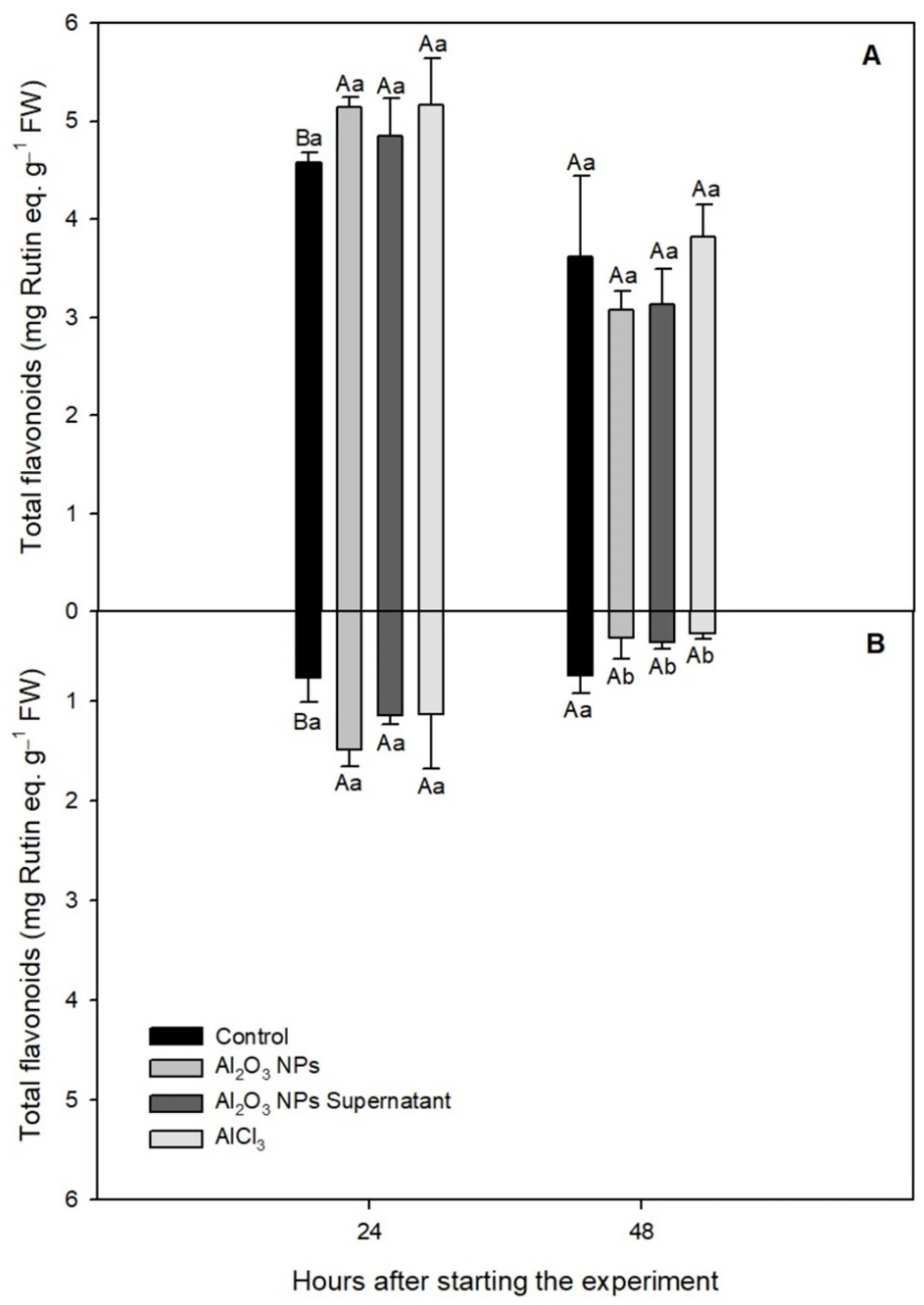
Total flavonoids in (**A**) leaves and (**B**) roots of perennial ryegrass (*L. perenne*) exposed to: (1) control (without NPs), (2) 500 mg L^−1^ Al_2_O_3_ NPs, (3) 500 mg L^−1^ Al_2_O_3_ NP supernatant, and (4) 200 μM AlCl_3_ for 24 and 48 h. Different capital letters indicate significant differences among treatments at the same exposure time according to Tukey’s test (*p* ≤ 0.05). Different lowercase letters indicate significant differences in exposure time (24 to 48 h) for the same treatment, as determined by the paired samples Student’s *t*-test (*p* ≤ 0.05).

Total flavonoid (TF) content in leaves was significantly higher in all treatments than in the control after 24 h of exposure, with no differences among the Al treatments (Figure 6A). However, after 48 h, no significant differences were detected among treatments. Temporal analysis showed that TF content remained unchanged in the control, whereas all Al treatments exhibited a significant decrease between 24 and 48 h.

A similar pattern was observed in roots (Figure 6B). At 24 h, all treatments showed significantly higher TF content than the control, while no differences among treatments were detected. After 48 h, TF levels no longer differed among treatments. As observed in leaves, TF content decreased significantly over time in all Al treatments, whereas the control remained unchanged. 

### 2.5. Lipid Peroxidation in L. perenne

**Figure 7:**
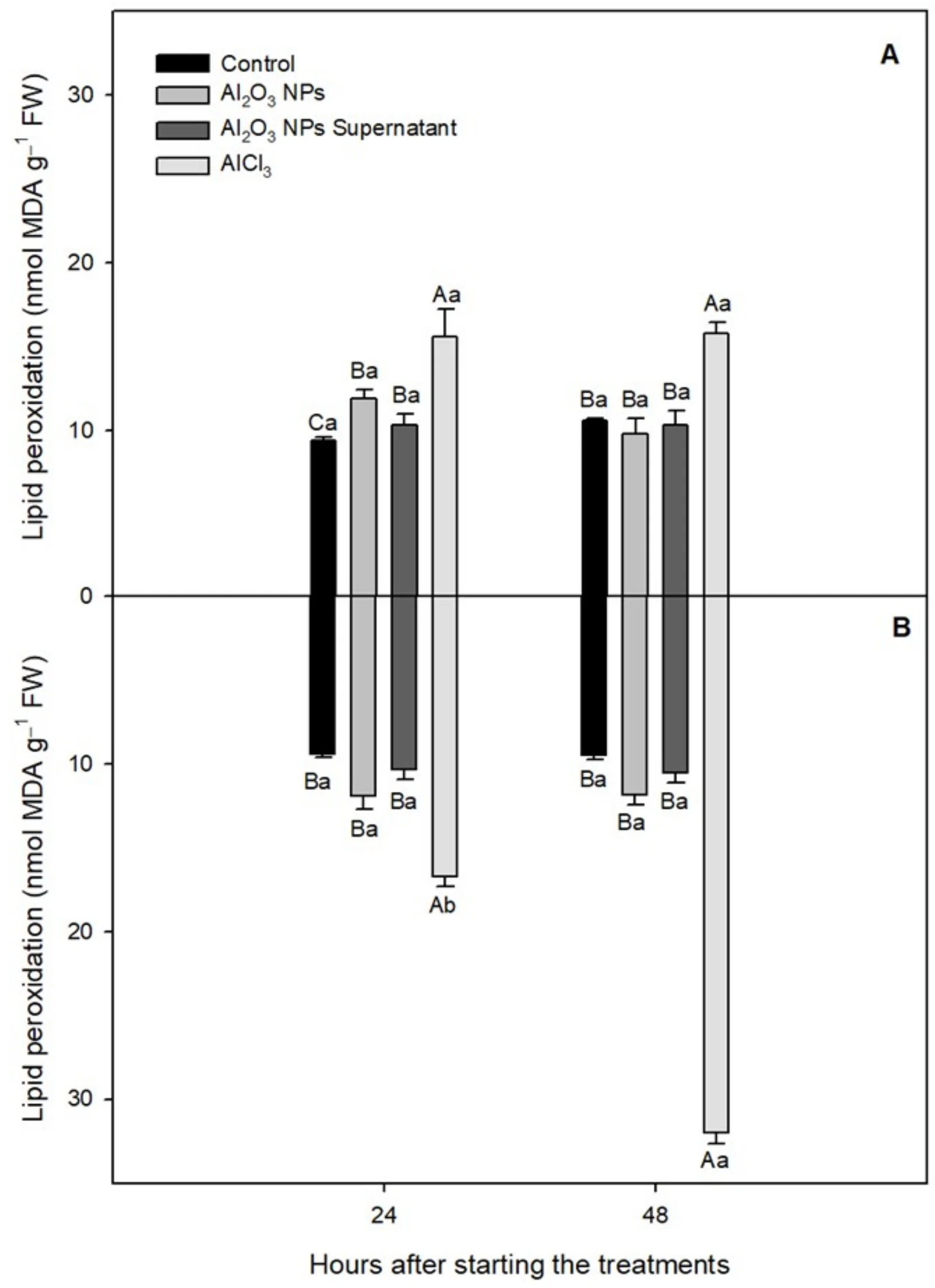
Lipid peroxidation in (**A**) leaves and (**B**) roots of perennial ryegrass (*L. perenne)* exposed to: (1) control (without NPs), (2) 500 mg L^−1^ Al_2_O_3_ NPs, (3) 500 mg L^−1^ Al_2_O_3_ NP supernatant, and (4) 200 μM AlCl_3_ for 24 and 48 h. Different capital letters indicate significant differences among treatments at the same exposure time according to Tukey’s test (*p* ≤ 0.05). Different lowercase letters indicate significant differences in exposure time (24 to 48 h) for the same treatment, as determined by the paired samples Student’s *t*-test (*p* ≤ 0.05).

Lipid peroxidation in leaves was significantly affected only by the AlCl_3_ treatment, which showed MDA levels approximately 1.6-fold higher than those of the control at both 24 and 48 h (Figure 7A). No significant differences were detected among the control, Al_2_O_3_ NPs, and Al_2_O_3_ NP supernatant treatments. In addition, no temporal changes in lipid peroxidation were observed in leaves for any treatment.

A similar pattern was observed in roots (Figure 7B), where AlCl_3_ also induced the highest lipid peroxidation at both exposure times. In contrast, the control, Al_2_O_3_ NPs, and Al_2_O_3_ NP supernatant treatments did not differ significantly. Temporal analysis revealed a significant increase in lipid peroxidation only in the AlCl_3_ treatment, which increased approximately 3.3-fold between 24 and 48 h. 

## 3. Discussion

In studies involving NPs, it is important to determine their average size and surface electrical characteristics in the medium in which they are applied to understand their interactions with ions and biological systems, as well as their state of aggregation [19]. For aluminum nanoparticles, these properties are significantly influenced by the medium’s pH [13,20]. The size and electrical charge of the NPs used in this study are consistent with those reported by Ghosh et al. [13] at pH 5, where the approximate size is > 300 nm, compared with 216.8 nm observed, and the Z potential is approximately +30 mV, compared with +32.7 mV observed in our work.

The Al_2_O_3_ NP supernatant and AlCl_3_ treatments produced more pronounced effects on several photosynthetic parameters than the Al_2_O_3_ NP suspension under the tested acidic conditions. Although CO_2_ assimilation and stomatal conductance decreased in all Al treatments, the Al_2_O_3_ NP supernatant and AlCl_3_ consistently produced the greatest reductions in ΦPSII, ETR, and gas exchange. This response agrees with the well-established predominance of soluble Al species under acidic conditions, where Al toxicity is recognized as one of the major constraints limiting photosynthesis and plant productivity [21], since Al_2_O_3_ dissolution increases as pH decreases [12]. These differences are consistent with a possible contribution of dissolved Al, but they do not establish its predominance as a driver of phytotoxicity. The observed responses cannot conclusively distinguish particle-mediated from dissolution-mediated effects because Al was not measured. 

At 48 h, Al_2_O_3_ NPs and AlCl_3_ reduced expression of *psbA* and *rbcS*, whereas *rbcL* expression did not differ significantly among treatments. Although *psbA* encodes the D1 protein of photosystem II [22], changes in its expression did not consistently align with the photochemical responses. Al_2_O_3_ NPs reduced *psbA* expression without significantly affecting ΦPSII or ETR, whereas the supernatant decreased both parameters without significantly altering *psbA* expression. These contrasting patterns indicate that *psbA* transcript abundance alone does not explain the photochemical responses across treatments. Although reduced *psbA* expression coincided with lower ΦPSII and ETR under AlCl_3_ exposure, this association does not establish a direct causal relationship.

Several studies have shown that the phytotoxicity of metal oxide nanoparticles depends not only on the particles themselves but also on their dissolution behavior in the exposure medium [23,24]. Under acidic or complexing conditions, dissolution can increase the release of soluble metal species, thereby enhancing biological responses [23,24]. Consistent with this, reductions in chlorophyll fluorescence parameters have been reported following exposure to different metal oxide nanoparticles [25,26,27,28,29]. For example, MgAl_2_O_4_ nanoparticles significantly reduced Fv/Fm, ΦPSII, and ETR in *Hordeum vulgare* [29], whereas similar alterations have been described for CuO and ZnO nanoparticles [26,27,28], supporting the view that disruption of PSII photochemistry is a common response to metal oxide nanoparticle exposure.

The simultaneous reduction in CO_2_ assimilation, ΦPSII, and ETR suggests that aluminum exposure promoted an imbalance between light energy absorption and carbon fixation, favoring the generation of reactive oxygen species (ROS) [30]. This interpretation is supported by the higher lipid peroxidation observed, particularly under AlCl_3_ treatment, indicating greater oxidative damage. Similar increases in lipid peroxidation and H_2_O_2_ accumulation have been reported in *Triticum aestivum* exposed to Al_2_O_3_ nanoparticles [31], reinforcing the association between aluminum exposure, oxidative stress, and impaired photosynthetic performance [32].

To cope with ROS and oxidative stress, plants activate antioxidant defense mechanisms, including biosynthesis of phenolic compounds such as phenols, flavonoids, and anthocyanins [33,34]. Additionally, phenolic compounds, through their complexing capacity, can modulate metal bioavailability, thereby buffering oxidative damage during Al and Cu exposure [35]. Consistent with these mechanisms, AA and TP increased in leaves and roots across all treatments, with the effect more evident at 48 h of exposure. Interestingly, Al_2_O_3_ NPs exhibited the highest TP level in leaves at 48 h, reaching values 2.5-fold higher than the control. Similarly, TF content increased significantly in leaves and roots at 24 h in all treatments, with no differences among them. In agreement with our findings, Yanik and Vardar [31] reported increased phenolic compound accumulation in *Triticum aestivum* exposed to low doses of Al_2_O_3_ NPs, whereas higher nanoparticle concentrations reduced phenolic levels, suggesting a dose-dependent antioxidant response. In our study, increased antioxidant activity and phenolic compound accumulation under Al_2_O_3_ NP and Al_2_O_3_ NP supernatant exposure were accompanied by lipid peroxidation levels that did not differ significantly from those of the control. These findings are consistent with a possible protective role of antioxidant responses; however, they do not establish a causal relationship between these responses and the limitation of oxidative damage. Such a protective mechanism remains a hypothesis requiring further experimental validation.

This study focused on short-term physiological and biochemical responses. Root growth and biomass were not assessed, and colloidal stability in Hoagland solution was not monitored during exposure. Dissolved Al concentrations and complete particle removal from the supernatant were also not verified. Addressing these aspects in future studies would help clarify the contributions of particulate and dissolved Al and their implications for plant growth.

## 4. Materials and Methods

### 4.1. Nanoparticle Characterization

Aluminum oxide nanoparticles (Al_2_O_3_ NPs) were purchased from Merck^®^ (product ID 544833, Merck KGaA, Darmstadt, Germany). The hydrodynamic size and zeta potential of the Al_2_O_3_ NPs were determined by dynamic light scattering (DLS) using a Zetasizer Nano ZS ZEN3600 (Malvern Instruments, Malvern, UK).

### 4.2. Plant Material and Growth Conditions

Seeds of *L. perenne* Halo-AR1 were germinated in six Petri dishes on filter paper moistened with 30 mL of deionized water. Seeds were disinfected with 1% sodium hypochlorite (NaClO) and distilled water before germination, resulting in a 92% germination rate. A total of 60 seeds were placed in each Petri dish and watered with calcium chloride (CaCl_2_) for 10 days. After 10 days, seedlings were transferred to 12 black 5-L pots, each containing 12 plants grown in Hoagland nutrient solution at pH 5.2 [36]. The experiment comprised four treatments, with three independent pots per treatment. Each pot constituted an experimental unit, whereas the plants within each pot constituted observational units. Thus, each treatment had three independent biological replicates (n = 3). Hoagland solution was renewed every 3 days, and the pH was adjusted to 5.2 every 2 days. Each pot was equipped with an aeration system connected to air pumps. Seedlings were grown under controlled conditions (16/8 h light/dark photoperiod, 24 ± 2°C, and 40% relative humidity) for 28 days for acclimation. Then, *L. perenne* seedlings were subjected to four treatments: control (without NPs), 500 mg L^−1^ Al_2_O_3_ NPs, 500 mg L^−1^ (according to Riahi-Madvar et al. [37] used in *Triticum aestivum*), Al_2_O_3_ NP supernatant, and 200 μM AlCl_3_ (according to Parra-Almuna [38]). The Al_2_O_3_ NP suspension was prepared at a nominal concentration of 500 mg L^−1^ and stirred for 30 min, sonicated for 30 min, and then stirred for an additional 30 min before application. The Al_2_O_3_ NP supernatant was obtained from the same procedure, but then centrifuged at 16,000× *g* for 10 min, similar to [39] for zinc NPs. At each evaluation time (24 and 48 h after the start of treatment), seven plants per pot were used for the determinations at 24 h, and the remaining seven plants were used at 48 h. Consequently, different plants were sampled at each exposure time, but both sampling times were represented within the same pots. Photosynthetic performance was assessed, and shoots and roots were then harvested from the same plants for gene expression and biochemical analyses. Samples were immediately frozen in liquid nitrogen and stored at −80°C, except those used in lipid peroxidation assays, which were stored at −20°C.

### 4.3. Chlorophyll Fluorescence of PSII and Photosynthetic Performance

Chlorophyll fluorescence measurements were determined as described by Reyes-Díaz et al. [40] using a portable pulse-amplitude-modulated fluorometer (FMS 2; Hansatech Instruments, King’s Lynn, UK). Before measuring Fv/Fm, fully expanded leaves were dark-adapted for 30 min using leaf clips equipped with a movable shutter plate to allow PSII reaction centers to return to the open state. Effective quantum yield (ΦPSII), electron transport rate (ETR), and non-photochemical quenching (NPQ) were determined as fluorescence parameters as described by Maxwell and Johnson [41]. The measurements were performed in the morning between 08:00 and 10:00 h. For gas exchange measurements, a portable infrared gas analyzer (IRGA) (Li-6400; LI-COR, Inc., Lincoln, NE, USA) was used, following the protocol of Reyes-Díaz et al. [42]. The analyzed parameters were CO_2_ assimilation (Pn), stomatal conductance (gs), and transpiration (E). The CO_2_ reference concentration was 400 μmol mol^−1^, with a flow rate of 300 mL min^−1^, a light intensity of 300 µmol m^−2^ s^−1^, 60% relative humidity, and a temperature of 20 ± 1°C. Chlorophyll fluorescence and gas exchange were measured in vivo during the light period from 08:00 to 10:00 h.

### 4.4. Gene Expression

#### 4.4.1. Total RNA Isolation and cDNA Synthesis

RNA was extracted using the rapid CTAB protocol developed by Gambino et al. [43] and modified by Inostroza-Blancheteau et al. [11]. To assess RNA integrity, samples were separated by electrophoresis in 1% agarose gels containing ethidium bromide at a final concentration of 0.5 µg mL^−1^, at 80 V for 45 min. RNA concentration was measured spectrophotometrically using an EPOCH^®^ microplate spectrophotometer (BioTek Instruments, Inc., Winooski, VT, USA) with a Take3^®^ microvolume plate (BioTek) and a Quantus^®^ dual-channel fluorometer (Cat. No. E6150) using a Promega QuantiFluor^®^ dye system (Cat. No. E3310). The RNA was treated with RNase-free DNase I (Thermo Fisher, Waltham, MA, USA) to eliminate genomic DNA contamination. For cDNA synthesis, 50 units of MultiScribe reverse transcriptase were used, following the instructions for the High-Capacity cDNA Reverse Transcription Kit (Thermo Fisher). Electrophoresis was subsequently performed in 1% agarose gels containing ethidium bromide at a final concentration of 0.5 µg mL^−1^, at 90 V for 30 min.

#### 4.4.2. Real-Time Quantitative PCR (qRT PCR) Analysis

The relative expression of the *psbA*, *rbcL*, and *rbcS* genes were analyzed using primers previously designed by Wu et al. [44] (see Table 2). *Actin* was used as the housekeeping gene. Real-time polymerase chain reactions (qPCR) were carried out using the GoTaq^®^ qPCR Master Mix kit from Promega^®^ (Madison, WI, USA), following the manufacturer’s instructions. Each 15 μL reaction contained 7.5 μL GoTaq^®^, 0.3 μL of each specific primer, and 3 μL of cDNA. The cycling conditions were as follows: 95°C for 10 min, followed by 40 cycles of 95°C for 15 s, 54 °C for 30 s, and 60 °C for 30 s. Relative gene expression was calculated using the comparative 2^(−ΔΔ) Ct method [45] with a StepOne™ Real-Time PCR System (Applied Biosystems, Foster City, CA, USA)—Table 2.

### 4.5. Photosynthetic Pigments Determination

The total chlorophyll *a* (Chl *a*) and chlorophyll *b* (Chl *b*) were analyzed in leaf samples of perennial ryegrass. Leaf samples (0.1 g) were ground in liquid nitrogen, macerated with ethanol (96% *v*/*v*), and centrifuged at 13,000× *g* for 5 min at 4°C, following the Lichtenthaler and Wellburn [46] protocol. The absorbance was measured at 665, 649, and 470 nm using a spectrophotometer (Thermo Scientific, GENESYS 10 UV, Rochester, NY, USA).

### 4.6. Determination of Antioxidant-Related Parameters

Antioxidant activity (AA), total phenols (TP), and total flavonoids were determined as antioxidant-related parameters in *L. perenne*. For AA and TP, 0.2 g leaf and root samples were ground in liquid nitrogen, macerated with 2 mL of ethanol (80% *v*/*v*), and centrifuged at 13,000× *g* for 10 min at 4 °C. The supernatant was collected and used for both determinations. Antioxidant activity was measured using the free radical 2,2-diphenyl-1-picrylhydrazyl (DPPH) according to the method described by Chinnici et al. [47]. The AA was measured at 515 nm using a spectrophotometer (Thermo Scientific, GENESYS 10 UV, Rochester, NY, USA) and expressed as µg of Trolox equivalent per gram of fresh weight (µg TE g^−1^ FW). For total phenols, the Folin–Ciocalteu method [48] was used, measuring absorbance at 765 nm. The results were expressed as µg of gallic acid equivalent per gram of fresh weight (µg GAE g^−1^ FW). Total flavonoids were determined by the aluminum chloride colorimetric method described by Cheng and Breen [49]. For this, leaf and root samples were macerated with methanol (80% *v*/*v*) and mixed with NaNO_2_ (5%) and distilled water. After 5 min of incubation, AlCl_3_ (10%) was added and incubated for 6 min. Then, NaOH (1 mol L^−1^) was added to the solution. Finally, the solution was incubated for 15 min. The absorbance was measured at 510 nm using the spectrophotometer (Thermo Scientific, GENESYS 10 UV, Rochester, NY, USA). Results were expressed as mg of rutin equivalents per g of fresh weight (mg Rutin g^−1^ FW).

### 4.7. Lipid Peroxidation of Msembranes

As an oxidative stress indicator, lipid peroxidation was measured in leaves and roots by quantifying thiobarbituric acid-reactive substances (TBARS). The absorbance was determined at 532, 600, and 440 nm to correct for TBARS-sugar interferences using a spectrophotometer (Thermo Scientific, GENESYS 10 UV, Rochester, NY, USA). The LP was expressed as malondialdehyde content equivalents per g of fresh weight (nmol MDA g^−1^ FW), following the modified method of Du and Bramlage [50].

### 4.8. Experimental Design and Data Analysis

A completely randomized block design with four treatments and three replicates was used. The experiment comprised four treatments, with three independent pots per treatment and 14 plants per pot, totaling 12 pots. Each pot constituted an experimental unit, and the plants within each pot were considered observational units. At 24 h, seven plants were harvested from each pot, and their tissues were pooled separately by tissue type to obtain one composite sample per pot for each tissue analyzed. The remaining seven plants were harvested and pooled in the same manner at 48 h. This procedure yielded three independent biological replicates per treatment at each sampling time (n = 3), with both sampling times represented within the same pots. Data were tested for normality and equal variance using the Shapiro–Wilk and Levene tests, respectively. Then, data were analyzed using one-way ANOVA, followed by a Tukey test for multiple comparisons (*p* ≤ 0.05) among treatments for the same exposure time. Paired samples Student’s *t*-test (*p* ≤ 0.05) was performed to compare exposure times for the same treatment. SigmaStat v. 2.0 (SPSS, Chicago, IL, USA) was used for statistical analysis.

## 5. Conclusions

This study demonstrates that aluminum exposure markedly impairs the physiological performance and photosynthetic capacity of *Lolium perenne* under acidic conditions. The strongest effects on gas exchange and PSII photochemical efficiency were observed in plants exposed to AlCl_3_ and the Al_2_O_3_ NP supernatant. However, the absence of dissolved Al measurements and verification of the particulate content of the supernatant precludes a definitive attribution of these effects to soluble Al. These physiological responses were accompanied by changes in the expression of photosynthesis-related genes and activation of antioxidant defense mechanisms. Although antioxidant activity and phenolic compound accumulation increased in response to aluminum exposure, these responses were insufficient to prevent oxidative damage under AlCl_3_ treatment. Further studies combining measurements of dissolved Al, verification of particle separation, and quantification of Al accumulation in plant tissues are needed to resolve the mechanisms underlying these treatment responses.

## Figures and Tables

**Table 1:** Concentration of photosynthetic pigments (mg g^−1^ FW) in perennial ryegrass (*L. perenne*) exposed to: (1) control (without NPs), (2) 500 mg L^−1^ Al_2_O_3_ NPs, (3) 500 mg L^−1^ Al_2_O_3_ NP supernatant, and (4) 200 μM AlCl_3_ for 24 and 48 h.

Treatments	Chl *a*	Chl *b*	Chl *a+b*	Chl *a/b*	Chl *a*	Chl *b*	Chl *a+b*	Chl *a/b*
	Exposure Time (h)							
	24				48			
Control	0.28 ± 0.01 Aa	0.11 ± 0.02 Aa	0.41 ± 0.01 Aa	2.44 ± 0.12 Aa	0.29 ± 0.01 Aa	0.12 ± 0.01 Ba	0.41 ± 0.02 ABa	2.74 ± 0.01 ABa
Al_2_O_3_ NPs	0.29 ± 0.01 Aa	0.09 ± 0.01 Aa	0.38 ± 0.02 Aa	3.18 ± 0.04 Aa	0.23 ± 0.01 Bb	0.06 ± 0.02 Ca	0.30 ± 0.01 Bb	4.04 ± 0.04 Aa
Al_2_O_3_ NPs S.	0.25 ± 0.02 Aa	0.07 ± 0.02 Ab	0.33 ± 0.05 Ab	4.00 ± 0.04 Aa	0.28 ± 0.01 Aa	0.19 ± 0.01 Aa	0.48 ± 0.06 Aa	1.83 ± 0.31 Bb
AlCl_3_	0.30 ± 0.01 Aa	0.11 ± 0.01 Aa	0.42 ± 0.02 Aa	2.70 ± 0.01 Aa	0.28 ± 0.05 Aa	0.08 ± 0.01 Ca	0.38 ± 0.01 ABa	3.20 ± 0.02 ABa

Different capital letters in the columns indicate significant differences among treatments at the same exposure time, as determined by Tukey’s test (*p* ≤ 0.05). Different lowercase letters in the rows indicate significant differences in the same treatment between the exposure times (24 and 48 h) according to the paired samples Student’s *t*-test (*p* ≤ 0.05). The values are the mean ± SE.

**Table 2:** Specific primers used for real-time PCR analysis to evaluate the expression of subunits of PSII and Rubisco.

Gene	Primers	
	Forward	Reverse
*psbA*	5′-TGGATGGTTTGGTGTTTTGATG-3′	5′-CCGTAAAGTAGAGACCCTGAAAC-3′
*rbcL*	5′-ATCTTGCTCGGGAAGGTAATG-3′	5′-TCTTTCCATACCTCACAAGCAG-3′
*rbcS*	5′-TGAGACTGAGCACGGATTTG-3′	5′-TTTAGCCTCTTGAACCTCAGC-3′
*Actin*	5′-GGATCTTGCTGGTCGTGATT-3′	5′-TCCTGGCAGTTTCAATCTCC-3′

## Data Availability

The original contributions presented in this study are included in the article. Further inquiries can be directed to the corresponding authors.

## Abbreviations

The following abbreviations are used in this manuscript:

AA	Antioxidant activity
Chl *a*	Chlorophyll *a*
Chl *b*	Chlorophyll *b*
E	Transpiration rate
ETR	Electron transport rate
Fv/Fm	Maximum photochemical efficiency of photosystem II
gs	Stomatal conductance
IRGA	Infrared gas analyzer
NPs	Nanoparticles
NPQ	Non-photochemical quenching
Pn	CO_2_ assimilation
PSII	Photosystem II
qPCR	Quantitative polymerase chain reaction
qRT-PCR	Real-time quantitative polymerase chain reaction
RNA	Ribonucleic acid
TF	Total flavonoid content
TP	Total phenol content
ΦPSII	Effective quantum yield of photosystem II

## References

[1] Nel A., Xia T., Mädler L., Li N. (2006). Toxic potential of materials at the nanolevel. Science.

[2] Mustafa G., Komatsu S. (2016). Toxicity of heavy metals and metal-containing nanoparticles on plants. Biochim. Biophys. Acta.

[3] Peters R., Bouwmeester H., Gottardo S., Amenta V., Arena M., Brandhoff P., Marvin H., Mech A., Moniz F., Pesudo L. (2016). Nanomaterial for products and application in agriculture, feed and food. Trends Food Sci. Technol..

[4] Ayenew B., Satheesh N., Zegeye Z., Kassie D. (2025). A review on the production of nanofertilizers and its application in agriculture. Heliyon.

[5] Shashidhara V., Bhattacharjee S., Alwarsamy M. (2025). Boosting Agronomic Yields with Metallic Nano-Fertilizers: An Effective Approach for Sustainable Agricultural Development. Commun. Soil Sci. Plant Anal..

[6] Miralles P., Church T., Harris A. (2012). Toxicity, Uptake, and Translocation of Engineered Nanomaterials in Vascular plants. Environ. Sci. Technol..

[7] Stadler T., Buteler M., Weaver D.K. (2010). Novel use of nanostructured alumina as an insecticide. Pest Manag. Sci..

[8] Remédios C., Rosário F., Bastos V. (2012). Environmental nanoparticles interactions with plants: Morphological, physiological, and genotoxic aspects. J. Bot..

[9] Kochian L.V., Pineros M.A., Hoekenga O.A. (2005). The physiology, genetics and molecular biology of plant aluminum resistance and toxicity. Plant Soil.

[10] Bernier R., Alfaro M. (2006). Acidez de los suelos y efecto del encalado. Bol. INIA.

[11] Inostroza-Blancheteau C., Reyes-Díaz M., Aquea F., Nunes-Nesi A., Alberdi M., Arce-Johnson P. (2011). Biochemical and molecular changes in response to aluminium-stress in highbush blueberry (*Vaccinium corymbosum* L.). Plant Physiol. Biochem..

[12] Furrer G., Stumm W. (1986). The coordination chemistry of weathering: I. Dissolution kinetics of *δ*-Al_2_O_3_ and BeO. Geochim. Cosmochim. Acta.

[13] Ghosh S., Mashayekhi H., Pan B., Bhowmik P., Xing B. (2008). Colloidal behavior of aluminum oxide nanoparticles as affected by pH and natural organic matter. Langmuir.

[14] Mora M.L., Alfaro M.A., Jarvis S.C., Demanet R., Cartes P. (2006). Soil aluminium availability in Andisols of Southern Chile and its effect on forage production and animal metabolism. Soil Use Manag..

[15] Pontigo S., Godoy K., Jiménez H., Guitérrez-Moraga A., Mora M.L., Cartes P. (2017). Silicon-Mediated alleviation of aluminum toxicity by modulation of Al/Si uptake and antioxidant performance in ryegrass plants. Front. Plant Sci..

[16] Parra-Almuna L., Pontigo S., Ruiz A., González F., Ferrol N., Mora M.D.l.L., Cartes P. (2024). Dissecting the Roles of Phosphorus Use Efficiency, Organic Acid Anions, and Aluminum-Responsive Genes under Aluminum Toxicity and Phosphorus Deficiency in Ryegrass Plants. Plants.

[17] Jin Y., Fan X., Li X., Zhang Z., Sun L., Fu Z., Lavoie M., Pan X., Qian H. (2017). Distinct physiological and molecular responses in *Arabidopsis thaliana* exposed to aluminum oxide nanoparticles and ionic aluminum. Environ. Pollut..

[18] Sánchez-Campos Y., Cárcamo-Fincheira P., González-Villagra J., Jorquera-Fontena E., Acevedo P., Soto-Cerda B., Nunes-Nesi A., Inostroza-Blancheteau C., Tighe-Neira R. (2023). Physiological and molecular effects of TiO_2_ nanoparticles application on UV-A radiation stress responses in *Solanum lycopersicum* L. Protoplasma.

[19] Bantz C., Koshkina O., Lang T., Galla H.J., Kirkpatrick C.J., Stauber R.H., Maskos M. (2014). The surface properties of nanoparticles determine the agglomeration state and the size of the particles under physiological conditions. Beilstein J. Nanotechnol..

[20] Missana T., Benedicto A., Mayordomo N., Alonso U. (2014). Analysis of anion adsorption effects on alumina nanoparticles stability. Appl. Geochem..

[21] Kochian L.V., Piñeros M.A., Liu J., Magalhaes J.V. (2015). Plant Adaptation to Acid Soils: The Molecular Basis for Crop Aluminum Resistance. Annu. Rev. Plant Biol..

[22] Theis J., Schroda M. (2016). Revisiting the photosystem II repair cycle. Plant Signal. Behav..

[23] Gao X., Avellan A., Laughton S., Vaidya R., Rodrigues S., Casman E., Lowry G. (2018). CuO Nanoparticle Dissolution and Toxicity to Wheat (*Triticum aestivum)* in Rhizosphere. Environ. Sci. Technol..

[24] Avramescu M.L., Chénier M., Beauchemin S., Rasmussen P. (2023). Dissolution Behaviour of Metal-Oxide Nanomaterials in Various Biological Media. Nanomaterials.

[25] Juhel G., Batisse E., Hugues Q., Daly D., van Pelt F.N., O’Halloran J., Jansen M.A. (2011). Alumina nanoparticles enhance growth of *Lemna minor*. Aquat. Toxicol..

[26] Dimkpa C.O., McLean J.E., Latta D.E., Manangón E., Britt D., Johnson W., Boyanov M., Anderson A. (2012). CuO and ZnO nanoparticles: Phytotoxicity, metal speciation, and induction of oxidative stress in sand-grown wheat. J. Nanopart. Res..

[27] Nair P.G.M., Chung I.M. (2014). A mechanistic study on the toxic effect of copper oxide nanoparticles in soybean (*Glycine max* L.) root development and lignification of root cells. Biol. Trace Elem. Res..

[28] Wang X., Yang X., Chen S., Li Q., Wang W., Hou C., Gao X., Wang L., Wang S. (2016). Zinc oxide nanoparticles affect biomass accumulation and photosynthesis in *Arabidopsis*. Front. Plant Sci..

[29] Alghofaili F., Tombuloglu H., Almessiere Alsaeed M., Tombuloglu G., Akhtar S., Baykal A., Veysi M., Turumtay H. (2025). Growth-Promoting and Genotoxic Effect of a Potential Nano Fertilizer (Magnesium Aluminum Oxide): A Double-Edged Sword. J. Soil Sci. Plant Nutr..

[30] Foyer C.H., Noctor G. (2003). Redox sensing and signaling associated with reactive oxygen in chloroplasts, peroxisomes and mitochondria. Physiol. Plant..

[31] Yanik F., Vardar F. (2015). Toxic Effects of Aluminum Oxide (Al_2_O_3_) Nanoparticles on Root Growth and Development in *Triticum aestivum*. Water Air Soil Pollut..

[32] Gill S.S., Tuteja N. (2010). Reactive oxygen species and antioxidant machinery in abiotic stress tolerance in crop plants. Plant Physiol. Biochem..

[33] Krause G.H., Weis E. (1991). Chlorophyll Fluorescence and Photosynthesis: The Basics. Annu. Rev. Plant Biol..

[34] González-Villagra J., Kurepin L.V., Reyes-Díaz M.M. (2017). Evaluating the involvement and interaction of abscisic acid and miRNA156 in the induction of anthocyanin biosynthesis in drought-stressed plants. Planta.

[35] Jańczak-Pieniążek M., Cichoński J., Michalik P., Chrzanowski G. (2023). Effect of Heavy Metal Stress on Phenolic Compounds Accumulation in Winter Wheat Plants. Molecules.

[36] Hoagland D., Arnon D. (1950). The water culture method for growing plants without soil. Calif. Agric. Exp. Stn. Univ. Calif..

[37] Riahi-Madvar A., Rezaee F., Jalali V. (2012). Effects of alumina nanoparticles on morphological properties and antioxidant system of *Triticum aestivum*. Iran. J. Plant Physiol..

[38] Parra-Almuna L., Diaz-Cortez A., Ferrol N., de la Luz Mora M. (2018). Aluminium toxicity and phosphate deficiency activates antioxidant systems and up-regulates expression of phosphate transporters gene in ryegrass (*Lolium perenne* L.) plants. Plant Physiol. Biochem..

[39] Heim J., Felder E., Tahir M.N., Kaltbeitzel A., Heinrich U.R., Brochhausen C., Mailänder V., Tremel W., Brieger J. (2015). Genotoxic effects of zinc oxide nanoparticles. Nanoscale.

[40] Reyes-Díaz M., Alberdi M., Mora M.L. (2009). Short-term aluminum stress differentially affects the photochemical efficiency of photosystem II in highbush blueberry genotypes. J. Am. Soc. Hortic. Sci..

[41] Maxwell K., Johnson G.N. (2000). Chlorophyll Fluorescence—A Practical Guide. J. Exp. Bot..

[42] Reyes-Díaz M., Inostroza-Blancheteau C., Millaleo R., Cruces E., Wulff-Zottele C., Alberdi M., Mora M. (2011). Long-term aluminium exposure effects on physiological and biochemical features of highbush blueberry cultivars. J. Am. Soc. Hortic. Sci..

[43] Gambino G., Perrone I., Gribaudo I. (2008). A rapid and effective method for RNA extraction from different tissues of grapevine and other woody plants. Phytochem. Anal..

[44] Wu Q., Su N., Shen W., Cui J. (2014). Analyzing photosynthetic activity and growth of *Solanum lycopersicum* seedlings exposed to different light qualities. Acta Physiol. Plant..

[45] Livak K.J., Schmittgen T.D. (2001). Analysis of relative gene expression data using real-time quantitative PCR and the 2^−ΔΔ^CT method. Methods.

[46] Lichtenthaler H., Wellburn A. (1983). Determinations of total carotenoids and chlorophylls a and b of leaf extracts in different solvents. Biochem. Soc. Trans..

[47] Chinnici F., Bendini A., Gaiani A., Riponi C. (2004). Radical scavenging activities of peels and pulps from cv. Golden Delicious apples as related to their phenolic composition. J. Agric. Food Chem..

[48] Singleton V.L., Rossi J.A. (1965). Colorimetry of total phenolics with phosphomolybdic-phosphotungstic acid reagents. Am. J. Enol. Vitic..

[49] Cheng G.W., Breen P.J. (1991). Activity of phenylalanine ammonialyase (PAL) and concentrations of anthocyanins and phenolics in developing strawberry fruit. J. Am. Soc. Hort. Sci..

[50] Du Z., Bramlage W. (1992). Modified thiobarbituric acid assay for measuring lipid oxidation in sugar-rich plant tissue extracts. J. Agric. Food Chem..

